# Sequence Analysis of Bitter Taste Receptor Gene Repertoires in Different Ruminant Species

**DOI:** 10.1371/journal.pone.0124933

**Published:** 2015-06-10

**Authors:** Ana Monteiro Ferreira, Andreia Tomás Marques, Mangesh Bhide, Vlatka Cubric-Curik, Kristin Hollung, Christopher Harold Knight, Katrine Raundrup, John Lippolis, Mitchell Palmer, Elvira Sales-Baptista, Susana Sousa Araújo, André Martinho de Almeida

**Affiliations:** 1 Instituto de Ciências Agrárias e Ambientais Mediterrânicas (ICAAM), Universidade de Évora, 7006–554 Évora, Portugal; 2 Plant Cell Biotechnology Laboratory, Instituto de Tecnologia Química e Biológica António Xavier (ITQB-UNL), Universidade Nova de Lisboa, 2780–157 Oeiras, Portugal; 3 Instituto de Investigação Científica Tropical, 1300–344 Lisboa, Portugal; 4 Laboratory of Biomedical Microbiology and Immunology, University of Veterinary and Pharmacy, 04181 Kosice, Slovakia; 5 University of Zagreb, Faculty of Agriculture, Department of Animal Science, 10000 Zagreb, Croatia; 6 NOFIMA, Norwegian Food Research Institute, N 1430 Aas, Norway; 7 Faculty of Health and Medical Sciences, University of Copenhagen, 2200 Copenhagen, Denmark; 8 Greenland Institute of Natural Resources, 3900 Nuuk, Greenland; 9 National Animal Disease Center, Ruminant Diseases and Immunology Research Unit, USDA, Ames, IA, 50010, United States of America; 10 National Animal Disease Center, Bacterial Diseases of Livestock Research Unit, USDA, Ames, IA, 50010, United States of America; 11 Departamento de Zootecnia, Universidade de Évora, 7002–554 Évora, Portugal; 12 CIISA—Centro Interdisciplinar de Investigação em Sanidade Animal, 1300–477 Lisboa, Portugal; 13 IBET-Instituto de Biologia Experimental e Tecnológica, 2780–157 Oeiras, Portugal; Duke University, UNITED STATES

## Abstract

Bitter taste has been extensively studied in mammalian species and is associated with sensitivity to toxins and with food choices that avoid dangerous substances in the diet. At the molecular level, bitter compounds are sensed by bitter taste receptor proteins (T2R) present at the surface of taste receptor cells in the gustatory papillae. Our work aims at exploring the phylogenetic relationships of T2R gene sequences within different ruminant species. To accomplish this goal, we gathered a collection of ruminant species with different feeding behaviors and for which no genome data is available: American bison, chamois, elk, European bison, fallow deer, goat, moose, mouflon, muskox, red deer, reindeer and white tailed deer. The herbivores chosen for this study belong to different taxonomic families and habitats, and hence, exhibit distinct foraging behaviors and diet preferences. We describe the first partial repertoires of T2R gene sequences for these species obtained by direct sequencing. We then consider the homology and evolutionary history of these receptors within this ruminant group, and whether it relates to feeding type classification, using MEGA software. Our results suggest that phylogenetic proximity of T2R genes corresponds more to the traditional taxonomic groups of the species rather than reflecting a categorization by feeding strategy.

## Introduction

The sense of taste is highly relevant for animal survival, as it probably evolved to provide animals with the ability to differentiate suitable from dangerous foods. There are five basic types of taste in mammals: sweet, salty, sour, bitter and *umami*. All act through a complex network of chemosensory receptors and signal transducers. Bitter taste iswell characterized for humans at both molecular and genetic levels, but little is known for ruminants, although they have the anatomical structures for taste perception and they make use of this important taste ability in their dietary choices [1–4]. Herbivores, ruminants included, have long been known to demonstrate preferences for different plant species and for parts of plants within species, so in a sense all herbivores are selective, maximizing nutrient intake and avoiding plant secondary metabolites [5]. According to the predominant type of feed ingested, herbivores can be classified into three feeding types: grazers (bulk and roughage feeders), browsers (selected diets containing at least 75% fruit, dicot foliage, and tree and shrub stems and foliage), and intermediate or mixed feeders (feeders that both browse and graze) [6].

Bitter taste has been extensively studied in various mammals and it is believed that it evolved to avoid the uptake of toxic substances, however, no strict correlation between bitterness and toxicity is observed [7]). At the molecular level, it is known that taste is sensed by taste receptor proteins present on the surface of taste receptor cells. Bitter taste receptors, in particular, are G-protein-coupled receptors (GPCRs) coded by a family of genes, TAS2R, that contain an average of 300 codons, and which are intronless. These characteristics make them easy to detect and analyze by DNA sequencing [3]. The repertoire of TAS2R (or T2R) is well described for several species, and shows rather large differences in gene numbers, from 15 genes in dogs to 54 in frogs, for example [2,3,8]. In the list of best (almost completely) described species are human, mouse [9,10], and chicken, consisting of 25, 34, and 3 functional genes, respectively [11,12]. Only scanty information on ruminant T2R genetics was available for cattle. Recently our team has reported eight T2R genes for sheep, applying a comparative genomics approach using cattle T2R data and the recently available sheep genome, followed by direct sequencing evidence using merino sheep DNA [13]. Using phylogenetic tools, we have also observed higher sequence conservation between the two ruminant species, sheep and cattle, than when comparing ruminants with other mammals.

In this present study, we have performed a larger sequence analysis to a collection of ruminant species belonging to different taxonomic families and habitats. Differrent foraging behaviors and diet preferences are represented within the group: five browsing species (fallow deer, moose, red deer, reindeer and white tailed deer), five grazing species (American and European bison, elk, mouflon, and sheep) and three intermediate feeding species (goats, musk ox and chamois). We aim to explore the phylogenetic relationships of T2R gene sequences within ruminant species. To achieve this goal, we described the first partial repertoire of T2R gene sequences for the chosen species and then studied the homology and phylogeny of these receptors within the ruminant group and in relation to previously described T2R sequences of non-ruminants.

## Materials and Methods

### Sampling and DNA extraction

We analysed samples of the following ruminant species: American bison (*Bison bison*), chamois (*Rupicapra rupicapra*), elk (*Cervus canadensis*), European bison (*Bison bonasus*), fallow deer (*Dama dama*), goat (*Capra hircus*), moose (*Alces alces*), mouflon (*Ovis ammon musimon*), muskox (*Ovibos moschatus*), reindeer (*Rangifer tarandus*), red deer (*Cervus elaphus*), sheep (*Ovis aries*) and white tailed deer (*Odocoileus virginianus*). Sheep and goat samples were obtained from animals at the University of Évora and the Faculty of Veterinary Medicine of the University of Lisbon, Portugal, respectively. Reindeer samples were obtained from the Beitostølen region in Norway. Red and fallow deer samples were obtained at the *Tapada de Mafra* Natural Reserve (Portugal). European bison samples were obtained from animals from the Tatra National Park in Slovakia, while chamois and mouflon samples were obtained respectively in the Biokovo Mountain and Sibenik region in Dalmatia, Croatia. Moose samples were obtained from animals in the Uppsala region in Sweden, muskox samples from the Kangerlussuaq region in Western Greenland, and American bison, elk and white tailed deer from the USDA experimental herd (Ames, IA, USA). DNA from one individual of each species was used in this study. Genomic DNA was isolated from blood samples, using the Qiagen DNeasy Blood & Tissue Kit (QIAGEN, Venlo, the Netherlands), according to the instructions by the manufacturer.

### Ethics statement

All the blood samples from which DNA was isolated were obtained during routine health monitoring, by specialized veterinary professionals. No animal experiment was performed; therefore, no specific ethical approval was necessary.

### PCR and sequencing

Using the Primer3 software version 0.4.0 (http://frodo.wi.mit.edu/), PCR primers (Table 1) were designed for seven T2R genes (*T2R3*, *T2R4*, *T2R10*, *T2R12*, *T2R13*, *T2R16*, *T2R67*) that we have previously found in sheep [13]. Amplification was optimized to be able to use one primer set only. T2R genes are intronless, so primers were designed on the exon sequence having the functional sheep T2R gene sequences as template, in order to amplify most of the coding sequence of each T2R gene (800–900bp). Oligonucleotides were synthesized by Stabvida (Stabvida, Caparica, Portugal). PCR reactions using approximately 75ng of DNA were carried out in a Bio-Rad C1000 Thermal Cycler (Bio-Rad Laboratories, Munich, Germany), using standard conditions, as previously described [13]. Sheep DNA was used as positive control. PCR products were loaded on a 1.5% agarose gel to confirm the existence of a unique band with the expected size. The PCR products were purified using the QIAquick PCR Purification Kit (Qiagen, Venlo, the Netherlands), following the instructions from the manufacturer. The purified PCR products were then analyzed by direct sequencing (Sanger method) as a purchased service from Stabvida (Stabvida, Caparica, Portugal) and using the same primer sets as for PCR reactions.

**Table 1:** Sequences of the primer sets used for each T2R gene amplification and sequencing.

Gene	Forward Primer Sequence 5'–3'	Reverse Primer Sequence 5'–3'
*T2R3*	AGCAATTTGGGGTTTCTGGT	TGGATAAACAGATCCCTTGGA
*T2R4*	TTTTTCTTCTATCGTTGTCTCTGAAA	TGCTTTTGTTTTCAGTTTAGGATG
*T2R10*	CAGTGGAAGGCCTCCTAATTT	TTCTCTTTTCCCCAGCACTT
*T2R12*	TGGAGAGAACACTGAACAATATACTTA	CATCACTTCAGGCTTATTTTTGG
*T2R13*	TGGCAGATTCTTTGGAAAACA	CACAGCACCAAAAGTGAAGC
*T2R16*	TGTCATAGTGCTGGGCAGAG	TCTTTTCAGTTTGGCACTGCT
*T2R67*	ATGCCATCTGGAATTGAAAA	TTTCAGGCGGCAGTTAAGAT

### Sequencing data analysis

Sequencing data were manually checked using Chromas Lite 2.1.1 (free at http://technelysium.com.au/) for visualization; FASTA files containing the DNA sequences were then used for conversion to protein sequences, using DNA to protein sequence converter at http://bioinformatics.picr.man.ac.uk/research/software/tools/sequenceconverter.html, and checked for ORFs using ORF Finder (http://www.ncbi.nlm.nih.gov/gorf/gorf.html). This application allowed us to check for premature stop codons that would indicate a pseudogene and for protein domains matching TAS2R protein family. Protein sequences in FASTA format were then used for multiple sequence alignment by Multiple Sequence Comparison by Log-Expectation (MUSCLE), freely available online at http://www.ebi.ac.uk/Tools/msa/muscle (version 3.8.31) [14,15]. Percent identity matrix was also produced by this software.

### Phylogenetic analyses

The MUSCLE data obtained in clustal format were used for phylogenetic analyses using MEGA version 6 software [16], available at http://www.megasoftware.net. Only protein sequences of intact genes were included. Sequences of pseudogenes were excluded, as previously described by Dong 2009 [3]. Similarly to Dong (2009), we also selected for the neighbor-joining statistical method of analysis and the bootstrap consensus tree was inferred from 500 replicates [17,18]. Evolutionary distances were computed using the JTT matrix-based method [19]. For a second phylogenetic analysis, protein sequences from T2R orthologues already reported for human and selected non-ruminant animal species of different trophic groups were used. These animal species were: chimpanzee (*Pan troglodytes*), dog (*Canis lupus*), horse (*Equus caballus*), mouse (*Mus musculus*), pig (*Sus scrofa*) and rabbit (*Oryctolagus cuniculus*). *Bos taurus* was included as reference ruminant species. The sequences were obtained either from Emsembl database (release 73—September 2013, http://www.ensembl.org), or, when not available, by using the GenBank to obtain DNA sequences and then convert them to protein sequence. These sequences were analyzed together with our sequencing data (converted to protein sequences) as input to the MEGA 6 software and the same analysis criteria were used as for the first analysis.

## Results

### T2R gene amplification and sequencing

PCR products of all seven genes analyzed were obtained for sheep, goat and mouflon, whereas, for the other species a lower number of genes were successfully amplified. PCR fragments obtained were of the expected length for all species using sheep fragments as control. Results of the PCR amplification of the seven different T2R analyzed in the 13 ruminant species are shown in Table 2. The sequencing results for each gene and species are shown in S1 Dataset. These sequences are also deposited in GeneBank (accession numbers KF898049-KF898092).

**Table 2:** Screening of T2R genes.

	*T2R3*	*T2R4*	*T2R10*	*T2R12*	*T2R13*	*T2R16*	*T2R67*
***Ovis aries***	+	+	+	+	+	+	+
***Capra hircus***	+	+	+	+	+	+	+
***Rangifer tarandus***	+	+	+	NP	+	NP	+
***Cervus elaphus***	NP	NP	+	NP	+	+	NP
***Dama dama***	+	NP	NP	+	+	NP	+
***Bison bonasus***	+	+	+	+	NP	+	NP
***Alces alces***	NP	NP	NP	NP	+	NP	+
***Ovis ammon musimon***	+	+	+	+	+	+	+
***Rupicapra rupicapra***	+	+	+	+	NP	+	NP
***Ovibos moschatus***	+	+	+	+	NP	+	+
***Bison bison***	NP	NP	+	+	+	+	NP
***Cervus canadensis***	+	+	NP	NP	NP	+	+
***Odocoileus virginianus***	+	+	+	+	NP	+	+

+ means successful amplification and sequencing.

NP means No PCR/Sequencing Product.

These sequences were then converted to protein sequences and a percent identity matrix produced, excluding pseudogenes to this analysis (matrix presented in S1 Table). The identities are grouped by receptor gene. The primers used in the study were able to amplify sequences ranging from 81–100% in similarity to the ovine genes, from which the sequences of primers were designed. For some of the genes we observed 100% matching of the gene in *Ovis ammon musimon* to *Ovis aries*.

### Phylogeny

**Fig 1:**
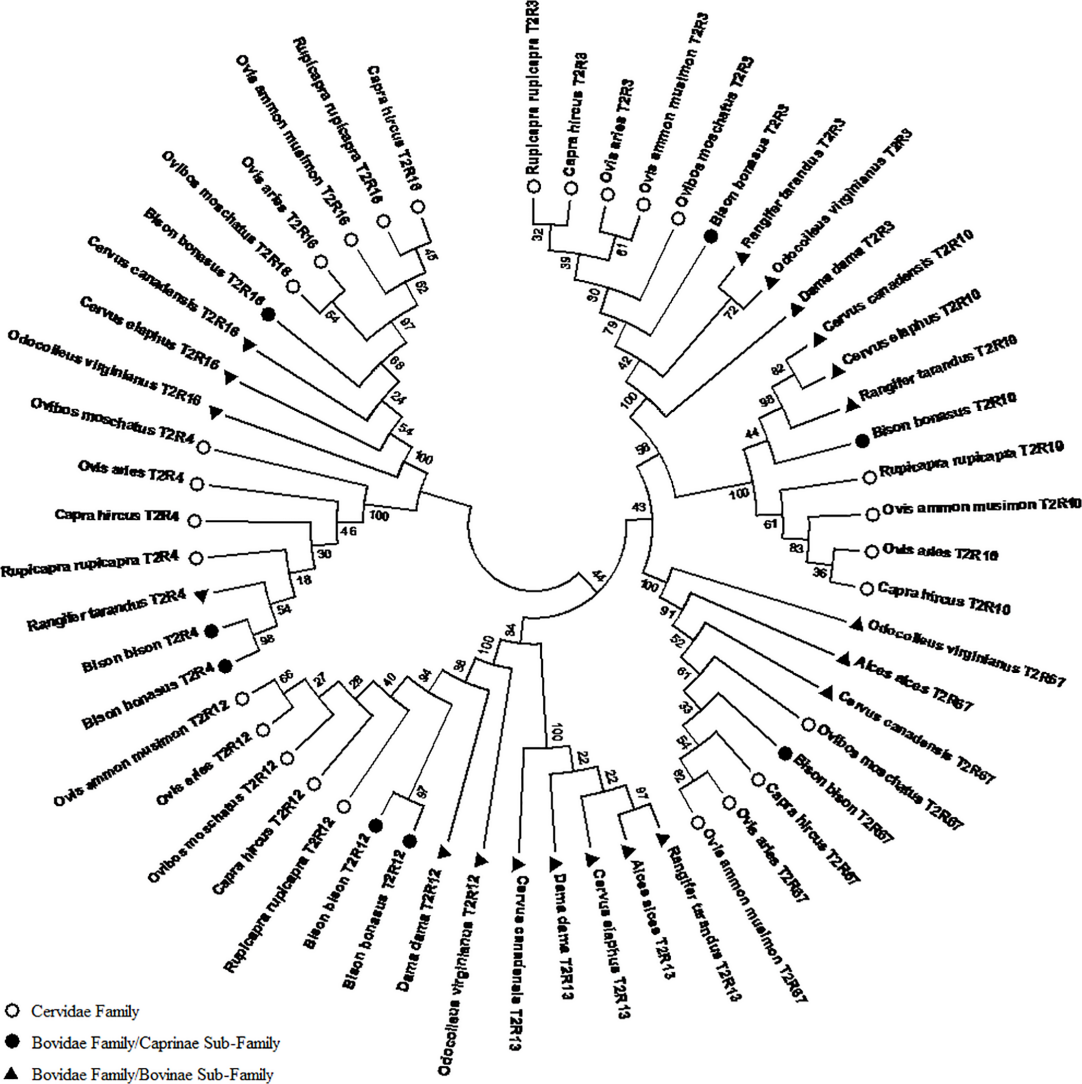
Phylogenetic tree built using MEGA software for the sequenced T2R genes in the different ruminant species. The evolutionary history was inferred using the Neighbor-Joining method [22]. The bootstrap consensus tree inferred from 500 replicates [21] is taken to represent the evolutionary history of the taxa analyzed [21]. Branches corresponding to partitions reproduced in less than 50% bootstrap replicates are collapsed. The evolutionary distances were computed using the JTT matrix-based method [23] and are in the units of the number of base substitutions per site. The analysis involved 55 amino acid sequences. All positions containing gaps and missing data were eliminated. There were a total of 96 positions in the final dataset. Evolutionary analyses were conducted in MEGA6 [20].

A phylogenetic tree was built with the protein sequences for the obtained intact genes, using Neighbor-joining statistical method of analysis, with a bootstrap value of 500 (Fig 1). There is evident clustering by receptor genes. We also observe that some receptors are closer to each other than others. For instance, T2R4 and T2R16 originate from the same branch, which is separated from the other branch in the root of two other sub-branches, one for receptors T2R3, T2R10, T2R67, and another for T2R13 and T2R12. The phylogenetic relations between species are not constant for every gene, nevertheless, there is a clear trend for phylogenetic distance or divergence of T2R genes to correspond to the traditional taxonomic groups of the species, rather than to feeding types (grazers, browsers or intermediate feeders). Species of the Bovidae family/Caprinae sub-family (sheep, mouflon, muskox, goat and chamois) form a cluster separated from species of the Bovidae family/Bovinae sub-family (American bison, European bison) and the Cervidae family (deer, elk, white tailed deer, reindeer, fallow deer and moose). Analyzing each receptor separately we can see different phylogenetic patterns, with the Cervidade family closer to the root of the branch, and Bovidae further away. However, some interesting exceptions are observed. For T2R10 species of the subfamily Bovinae are closer to the Cervidae family than to the Caprinae sub-family of their own family (Bovidae), and for T2R67, there appears to exist a divergence for each species at a time, not in clusters, albeit keeping the same taxonomic proximities. Finally, *O*. *aries*, does not cluster with *O*. *ammon musimon* for every gene even though they are of the same genus. For example, in T2R10, *O*. *aries* is closer to *C*. *hircus*, or even to *O*. *moschatus* for receptor gene T2R16. Another interesting finding was that for T2R13 we were only able to find intact genes in the Cervidae samples. We could successfully amplify and sequence PCR fragments for other species but the resulting sequences have premature stop codons, indicatig pseudogenezation of this gene for those species.

A second phylogenetic analysis was performed, extending the comparison with the sequencing data published for the orthologous genes for other animals with even more different feeding strategies, including non-ruminants and within distant taxa (S1 Fig). S2 Table shows the alignment of the 103 sequences used and the 80 residues included in the final analyses, when excluding gaps and missing data. The correlation of the T2R genes grouping with the taxonomic relationships was maintained. For example, cattle clustered with American and European bison at all genes for which the sequencing data was available, confirming also this grouping of Bovinae at one branch.

## Discussion

In the present study a sequence analysis of T2R genes is presented for the first time for a wide collection of ruminant species. Most of these species have very little genomic data available and we confirm that the approach herein described and developed using data available from other sequenced ruminants is a good comparative strategy to find missing genetic information; at least for features that are coded by considerably conserved genes, such as genes within the bitter taste receptor family. This is in accordance with previous genetic linkage mapping studies made with ruminants and humans [20,21]. In addition to obtaining the DNA sequence of several T2R we also studied the homology of T2R genes within the ruminant animal group and compared it to non-ruminant species, in order to study possible correlations with the different dietary habits of the different species. Our results show more evidence for phylogenetic distance or divergence of T2R genes corresponding to the traditional taxonomic groups of the species, rather than to the feeding types of these ruminants (grazers, browsers or intermediate feeders).

Novel sequences for T2R genes are presented for the following ruminant species, building their first partial repertoire of T2R: *A*. *alces*, *B*. *bison*, *B*. *bonasus*, *C*. *hircus*, *C*. *canadensis*, *C*. *elaphus*, *D*. *dama*, *O*. *virginianus*, *O*. *ammon musimon*, *O*. *moschatus*, *R*. *tarandus* and *R*. *rupicapra*. The protein sequence identities among species for each sequenced receptor ranged from 81 to 100%, confirming evident clustering by receptor gene. The results can be also related to the fact that the same sheep gene-based primer sets were used for all species. Possibly genes with lower similarity levels, or with high similarities but containing gaps in the template sequence at the annealing point of the primers, could not be amplified by PCR and, therefore, were not selected for sequencing. We also cannot discard the possibility that some species might simply not have some receptors, as we know that different animal species have different number of T2R and/or different proportions of genes/pseudogenes in their T2R repertoires [2,4]. Interestingly for T2R13 we were only able to find intact genes for the Cervidae samples. We successfully amplified and sequenced PCR fragments for other species but the resulting sequences were pseudogenes. It is well known that some animal species have different number of T2R and/or different proportions of genes/pseudogenes in their T2R repertoires [2,4]. This result also suggests that this receptor in particular may not be necessary to function in some environments or for some species which are not sensitive to the bitter compounds perceived through this receptor.

In terms of phylogenetic relations between the sequences obtained, we observed that there is a correlation between taxonomic classification of the species studied and the obtained receptor sequences, following the general proximity levels of their genomes, i.e., from a phylogenetic analysis the divergence of species of the Bovidae family/Caprinae sub-family (sheep, mouflon, goat, muskox and chamois) from species of the Bovidae family/Bovinae sub-family (American and European bison) and from the Cervidae family (red deer, reindeer, fallow deer,moose, elk and white tailed deer) became obvious. However, we could find exceptions. For T2R10, species of the subfamily Bovinae are closer to the Cervidae family than to the Caprinae sub-family of the same (Bovidae) family. Also *O*. *aries* does not cluster with *O*. *ammon musimon* for every gene: for T2R10 *O*. *aries* is closer to *C*. *hircus*, for T2R16 to *O*. *moschatus*. We propose that these small alterations might be related to differences in diet or similarities in the environment and/or available food types, more specifically for the bitter substances that are recognized by those particular receptors. A database of bitter taste receptors and corresponding ligands has been built for humans [22]. Such information is not yet available for all these ruminants, thus further studies are needed to predict what food types are more responsible for these differences. However, it is noteworthy that recent studies have shown that in humans very similar receptors may have highly divergent agonist spectra [23,24], whereas dissimilar receptors can have considerable overlaps in their activating bitter compounds [25]. Therefore, sequence similarity level cannot accurately predict for TAS2R specificity. This might explain why we were not able to find a correlation between T2R gene similarity and feeding strategies of the different animals studied. Interestingly, the same correlation of the T2R genes grouping by taxonomic positions was maintained when previously reported sequences of T2R from mammals other than ruminants were introduced to the analyses. For instance, *B*. *taurus* genes, the main reference ruminant species for the known T2R gene repertoire, confirmed our phylogenetic results by consistently clustering with *B*. *bison* and *B*. *bonasus*.

As previously referred to in more extensively studied species, there is an ongoing evolutionary diversification of T2R receptors [26], where differences found in the T2R gene sequences and, therefore, protein sequences among species are likely related with the need to adapt to an environment with a different diet, and consequently food choices. Our study extends this hypothesis to different trophic groups of herbivores. However, we could not find a strong correlation of the sequence similarities with the feeding category of each ruminant (grazers, browsers or intermediate feeder), at least not stronger than the clustering by taxa. For instance, sheep are closer to goat in gene sequences than to European bison, but sheep and European bison are grazers while goat is an intermediate feeder. It is important to keep in mind that feeding types are indicative of feeding habits and that they can have no relationship with the actual content of bitter substances in the feeds. Ruminant evolution may be classified taking the physical and mechanical characteristics of the respective forages into account [27], where intake is related to relative forestomach capacity, and body weight. This classification is not without exceptions. For instance, muskox and reindeer cannot be placed into categories with other species. They have particular feeding habits, as they are adapted to survive and reproduce under the severe constraints of the Arctic. Muskox prefer a diet of graminoids, sedges and dicots [28] whereas reindeer prefer lichens [29]. Such different food sources certainly contain different bitter ligands for different T2R receptors.

We hypothesize that other genetic differences exist, for example at the level of number of genes or ratio of pseudogenes/funtional genes, and are not reflected at the level of ortholog functional genes. The number of (functional) T2R genes seems to be important for taste perception and it has been proposed that carnivores have fewer T2R genes, herbivores an intermediate number, and omnivores the largest T2R gene repertoire [30]. The number of taste genes has also been addressed for other taste receptors in carnivorous mammals. Jiang *et al*. (2012) have found pseudogenized TAS1R genes and suggested that T2R losses are consistent with altered feeding strategies when they could not detect intact T2R genes in the dolphin genome [31]. Sato & Wolsan (2012), on the other hand, recently hypothesized that factors underlying the pseudogenization of TAS1R1 in pinnipeds may be driven by the specific marine environment to which these animals are adapted, namely the feeding behavior of swallowing food whole without mastication (T1R1 + T1R3 receptor is distributed on the tongue and palate), and the saltiness of sea water (a high concentration of sodium chloride masks *umami* taste) [32]. Moreover, Li & Zhang (2014) have also proposed that the number of T2R genes in a species correlates with the fraction of plants in its diet, and supported the hypothesis that dietary toxins are the driving force behind the differences in T2R repertoires among species [33]. However, we can only speculate on the number of genes as, with the strategy used in this study, only a portion of the total repertoire of T2R genes of each species is unraveled and compared. For other taste receptors, it has been shown that function can be obtained using alternative strategies when a certain receptor is absent from the genome. For instance, in 2014 Baldwin and colleagues working in hummingbirds demonstrated that the widespread absence from birds of an essential subunit (T1R2) of the only known vertebrate sweet receptor can be substituted by the ancestral umami receptor T1R1-T1R3 heterodimer [34]. Behrens et al. (2014) have also addressed similar problems in birds, demonstrating that the small TAS2R gene repertoire of chicken and turkey compensates low gene number by large tuning breadths [12].

We demonstrate that using PCR primers from a related species within the same trophic group is a good strategy to find T2R genes in species for which the genomes are not yet available. Nevertheless, a strategy using degenerate primers could be used in future works to further complement the present repertoire. The knowledge of these sequences may also be helpful to understand the taste perception mechanisms in these animals, particularly if expression studies will follow. Moreover, data on bitter taste perception in ruminants may have a high impact in animal nutrition with important consequences for the optimization of feed utilization, hence contributing to more sustainable and efficient ruminant production systems.

## Supporting Information

S1 Dataset DNA sequences obtained for each T2R gene and species, in FASTA format. Species: sheep (Ovis aries), goat (Capra hircus, reindeer (Rangifer tarandus, red deer (Cervus elaphus), fallow deer (Dama dama), European bison (Bison bonasus), moose (Alces alces), chamois (Rupicapra rupicapra), mouflon (Ovis ammon musimon), muskox (Ovibos moschatus), American bison (Bison bison), white tailed deer (Odocoileus virginianus) and elk (Cervus canadensis).
(TXT)

S1 Fig Phylogenetic tree obtained with the sequencing data of T2R genes on our ruminants and the orthologous genes for other animals including non-ruminants, using MEGA software. Protein sequences were used for building the tree. The evolutionary history was inferred using the Neighbor-Joining method. The bootstrap consensus tree inferred from 500 replicates is taken to represent the evolutionary history of the taxa analyzed. Branches corresponding to partitions reproduced in less than 50% bootstrap replicates are collapsed. The evolutionary distances were computed using the JTT matrix-based method and are in the units of the number of amino acid substitutions per site. The analysis involved 103 amino acid sequences. All positions containing gaps and missing data were eliminated. There were a total of 80 positions in the final dataset. Evolutionary analyses were conducted in MEGA6.
(PDF)

S1 Table Percent identity matrix. (XLS)

S2 Table Scheme of the alignment of the 103 sequences and the 80 residues included in the final analyses. (XLS)
