# Supplementary figures and images for: Sequence Analysis of Bitter Taste Receptor Gene Repertoires in Different Ruminant Species
Source: PLoS One. 2015 Jun 10;10(6):e0124933. doi: 10.1371/journal.pone.0124933 (PMC4465170; doi:10.1371/journal.pone.0124933)

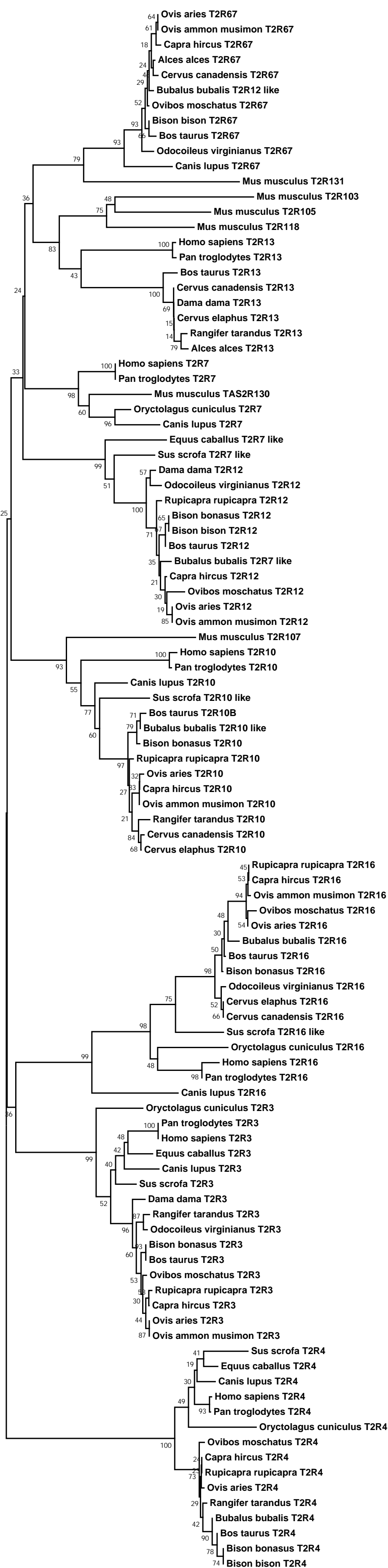

0.2

Supplement: S1 Fig — Protein sequences were used for building the tree. The evolutionary history was inferred using the Neighbor-Joining method. The bootstrap consensus tree inferred from 500 replicates is taken to represent the evolutionary history of the taxa analyzed. Branches corresponding to partitions reproduced in less than 50% bootstrap replicates are collapsed. The evolutionary distances were computed using the JTT matrix-based method and are in the units of the number of amino acid substitutions per site. The analysis involved 103 amino acid sequences. All positions containing gaps and missing data were eliminated. There were a total of 80 positions in the final dataset. Evolutionary analyses were conducted in MEGA6. (PDF) [file pone.0124933.s002.pdf]
